# Crystal structure of (methanol-κ*O*)[5,10,15,20-tetra­kis­(2-amino­phen­yl)porphyrinato-κ^4^ *N*]zinc(II)–chloro­form–methanol (1/1/1)

**DOI:** 10.1107/S205698901801099X

**Published:** 2018-08-21

**Authors:** Lisa Leben, Christian Näther, Rainer Herges

**Affiliations:** a Institut für Organische Chemie, Universität Kiel, Otto-Hahn-Platz 4, 24118, Kiel, Germany; b Institut für Anorganische Chemie, Universität Kiel, Otto-Hahn-Platz 6/7, 24118, Kiel, Germany

**Keywords:** crystal structure, picket fence porphyrin, atropisomers, zinc(II) porphyrin

## Abstract

In the crystal structure of the title compound, [Zn(C_44_H_32_N_8_)(CH_3_OH)]·CHCl_3_·CH_3_OH, the Zn^II^ cation is coordinated by four porphyrin N and one methanol O atom within a slightly distorted square-pyramidal environment and is shifted out of the porphyrin plane towards the direction of the methanol mol­ecule. The methyl group of the coordinating methanol mol­ecule is disordered over two sets of sites. The porphyrin backbone is nearly planar and the phenyl rings are almost perpendicular to the porphyrin plane. As is typical for picket-fence porphyrins, all four *ortho* substituents of the *meso*-phenyl groups (here the amino groups) are facing to the same side of the porphyrin mol­ecule. In the crystal structure, two neighbouring porphyrin complexes form centrosymmetric dimers that are connected *via* O—H⋯N hydrogen bonding. With the aid of additional N—H⋯N and C—H⋯N hydrogen bonding, these dimers are stacked into columns parallel to [010] that are finally arranged into layers parallel to (001). Between these layers channels are formed where chloro­form solvent mol­ecules are located that are connected to the porphyrin complexes by weak C—H⋯Cl hydrogen bonding. There are additional cavities in the structure where some small residual electron density is found, indicating the presence of disordered methanol mol­ecules, but a reasonable model could not be refined. Therefore the contribution of the electron density associated with the methanol solvent mol­ecule was removed with the SQUEEZE procedure [Spek (2015 ▸). *Acta Cryst.* C**71**, 9–18] in *PLATON*. Nevertheless, the given chemical formula and other crystal data take into account the methanol solvent mol­ecule.

## Abstract 2

The synthesis and crystal structure of 5,10,15,20-tetra­kis α,α,α,α 2-amino­phenyl zinc(II) porphyrin is reported, which is a potent starting material for the synthesis of several other picket fence porphyrins.

## Chemical context   

**Figure 1:**
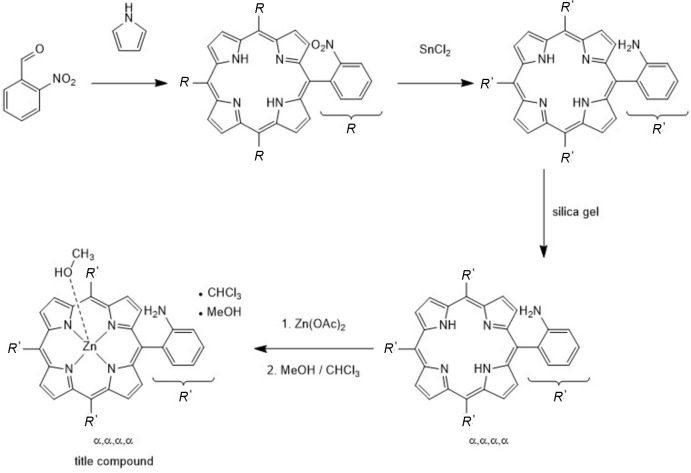
Reaction scheme for the synthesis of the title compound 5,10,15,20-tetra­kis α,α,α,α 2-amino­phenyl zinc(II) porphyrin.

Picket-fence porphyrins have been widely used as model compounds for the investigation of oxygen binding to hemoproteins (Collman *et al.*, 1975 ▸, 1976 ▸; Tabushi *et al.*, 1985 ▸; Schappacher *et al.*, 1989 ▸). With bulky substituents in the *ortho*-positions of the *meso-*substituents, their rotation is hindered, leading to only one side of the porphyrin being accessible for axial coordination in the all-α isomer. In 1973, Collman *et al.* for the first time reported this behaviour on the prototype picket-fence porphyrin 5,10,15,20-tetra­kis α,α,α,α 2-pivala­mido­phenyl porphyrin (Collman *et al.*, 1973 ▸). Afterwards, the first crystal structure of a picket-fence porphyrin was published (Collman *et al.*, 1975 ▸). Since that time, several different substituted picket-fence porphyrins have been reported (Collman *et al.*, 1983 ▸, 1998 ▸; Lee *et al.*, 2010 ▸; Yu *et al.*, 2015 ▸). In general, there is a risk of isomerization to the other atropisomers, but with the incorporation of zinc(II) the rotational barrier for the *meso*-substituents is increased, as reported by Freitag & Whitten (1983 ▸). Therefore, harsher reaction conditions could be used to introduce substituents in the *ortho*-positions without atropisomerization. We became inter­ested in this class of compounds as receptors for oxo anions. We synthesized the title compound in a four-step synthesis using 2-nitro­benzaldehyde and pyrrole as starting material (Fig. 1 ▸) as the key precursor for further functionalizations. Surprisingly, no crystal structure of this compound has been reported. We inserted Zn^II^ into the porphyrin to stabilize its planar geometry and thus to prevent atropisomerization. Single crystals could be obtained from a methanol/chloroform solution of the zinc(II) porphyrin complex, and were characterized by single-crystal X-ray diffraction.
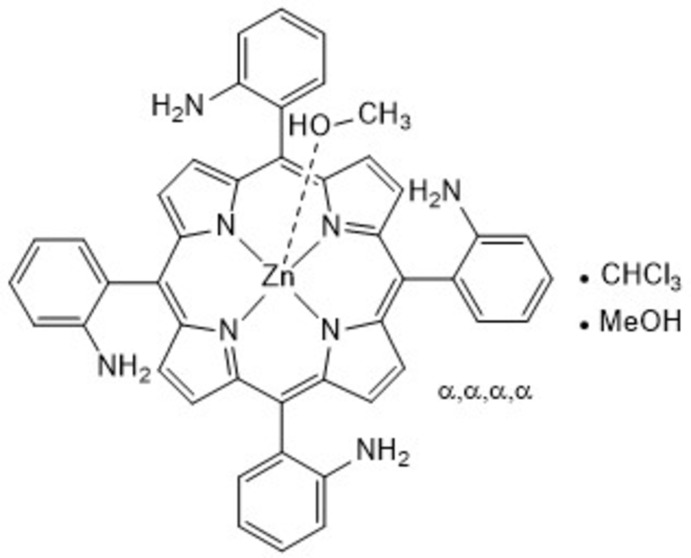


## Structural commentary   

**Figure 2:**
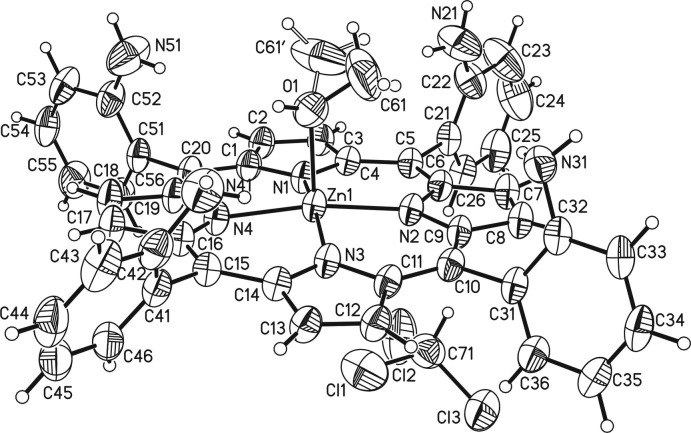
The structure of the mol­ecular entities in the title compound with labelling and displacement elliposids drawn at the 50% probability level. The disorder of the methyl group is shown as full and open bonds.

The asymmetric unit of the solvated title compound, [Zn(C_44_H_32_N_8_)(CH_3_OH)]·CHCl_3_·CH_3_OH, consists of one Zn^II^ cation, one substituted porphyrin, one methanol, as well as one chloro­form solvent mol­ecule, all of them located in general positions (Fig. 2 ▸). The contribution of an additional methanol solvent mol­ecule to the electron density was removed with the SQUEEZE procedure in *PLATON* (Spek, 2015 ▸). The methyl group of the methanol mol­ecule is disordered over two positions and was refined using a split model. All four amino groups are located on the same side of the porphyrin moiety, which shows that the α,α,α,α isomer was obtained. The porphyrin backbone is nearly planar, the largest deviation from the mean least-squares plane amounts to 0.189 (3) Å. All phenyl rings are nearly perpendicular to the porphyrin plane, with dihedral angles of 85.86 (9), 74.90 (7), 67.75 (6) and 85.17 (7)°.

**Figure 3:**
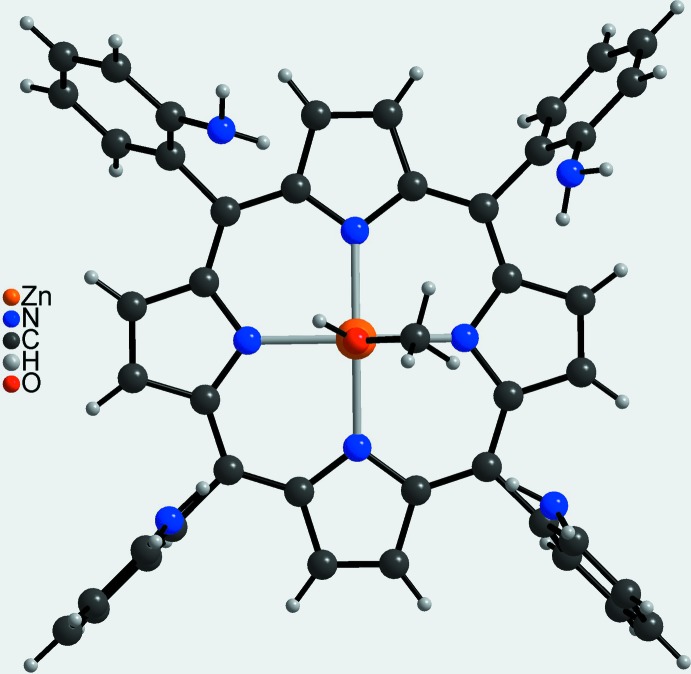
Mol­ecular structure of a discrete complex in a view into the porphyrin plane. The disordered methyl group is shown with the major component.

The zinc(II) cation is coordinated by four porphyrin N atoms that are located in the basal plane, and the metal coordination is completed by the O atom of a methanol mol­ecule in apical position leading to an overall square-pyramidal environment (Fig. 3 ▸). The Zn—N distances range from 2.050 (2) to 2.060 (2) Å and correspond to literature values (Table 1 ▸). As expected, the apical Zn—O distance of 2.143 (2) Å is slightly longer (Table 1 ▸). All angles around the Zn^II^ cation scatter between 88.96 (8) and 89.73 (8)° for basal groups and between 92.93 (8) and 98.66 (9)° involving the apical group, which shows that the coordination polyhedron is slightly distorted (Table 1 ▸). The Zn^II^ cation is located 0.1876 (9) Å above the mean plane formed by Zn1, N1, N2, N3 and N4 and is shifted towards the direction of the methanol O atom.

## Supra­molecular features   

**Figure 4:**
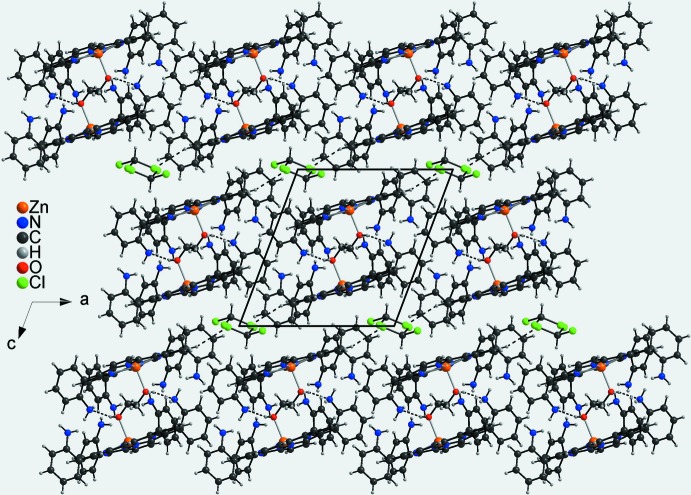
Crystal structure of the title compound in a view along [010]. Inter­molecular O—H⋯N and C—H⋯Cl hydrogen bonds are shown as dashed lines.

In the crystal structure of the title compound, each two neighbouring porphyrin complexes form dimers that are located on centers of inversion. The methanol mol­ecules are directed into the cavity of the dimer and are linked to the symmetry-related complex by inter­molecular O—H⋯N hydrogen bonding (Fig. 4 ▸, Table 2 ▸). These dimers are stacked into columns extending parallel to [001] (Fig. 4 ▸). The columns are connected by weak N—H⋯N and additional C—H⋯N inter­actions into layers parallel to (001). Between the layers channels are formed, in which the chloro­fom solvate mol­ecules are embedded. The solvent mol­ecules are linked to the porpyhrine complexes by inter­molecular C—H⋯Cl hydrogen bonding (Fig. 4 ▸, Table 2 ▸).

## Database survey   

In 1975, Collman *et al.* determined the first crystal structure of a picket-fence porphyrin (Collman *et al.*, 1975 ▸). In the past decades, numerous other crystal structures of picket-fence porphyrins have been published (Nasri *et al.*, 1987 ▸; Collman *et al.*, 1988 ▸; Michaudet *et al.*, 2000 ▸; Zimmer *et al.*, 2002 ▸; Ruzié *et al.*, 2006 ▸; Li *et al.*, 2013 ▸). For the α,β,α,β isomer of tetra­kis 2-amino­phenyl porphyrin, a crystal structure was published by Zimmer *et al.* (2002 ▸). A crystal structure for the tetra­kis α,α,α,α 2-amino­phenyl porphyrin has not been reported so far.

## Synthesis and crystallization   

The metal-free 5,10,15,20-tetra­kis α,α,α,α 2-amino­phenyl porphyrin was synthesized according to procedures reported by Collman *et al.* (1975 ▸) and Lindsey (1980 ▸). For the insertion of zinc(II), standard metallation conditions were used (Strohmeier *et al.*, 1997 ▸): 5,10,15,20-tetra­kis α,α,α,α 2-amino­phenyl porphyrin (30 mg, 44 mmol), zinc(II) acetate dihydrate (195 mg, 889 mmol) and 0.5 ml tri­ethyl­amine were stirred in 10 ml of di­chloro­methane for 24 h at room temperature. The reaction mixture was washed with water (2 × 30 ml) and dried over magnesium sulfate. After flash coloumn chromatography (cyclo­hexane / ethyl acetate, 20 to 100% ethyl acetate) 30 mg (41 mmol; 92% yield) of 5,10,15,20-tetra­kis α,α,α,α 2-amino­phenyl zinc(II) porphyrin were obtained. For crystallization, the compound was dissolved in chloro­form and precipitated with methanol.

^1^H NMR (500 MHz, DMSO-*d*_6_, 300 K): δ = 8.74 (*s*, 8H, H-β), 7.68 (*dd*, ^3^ *J* = 7.4 Hz, ^4^ *J* = 1.5 Hz, 4H, H-6), 7.50 (ddd, ^3^ *J* = 8.1, 7.6 Hz, ^4^ *J* = 1.6 Hz, 4H, H-4), 7.13 (*dd*, ^3^ *J* = 8.3 Hz, ^4^ *J* = 1.0 Hz, 4H, H-5), 7.00 (*dt*, ^3^ *J* = 7.4 Hz, ^4^ *J* = 1.0 Hz, 4H, H-3), 4.43 (*s*, 8H, NH) ppm. ^13^C NMR (125 MHz, DMSO-*d*_6_, 300 K): δ = 149.5 (C-α), 147.9 (C2), 134.3 (C6), 131.2 (C-β), 128.8 (C4), 126.8 (C1), 116.1 (C-*meso*), 115.4 (C5), 114.5 (C3) ppm. EI–MS: *m*/*z* (%) = 736.2 (100) [*M*]^+^.

## Refinement   

Crystal data, data collection and structure refinement details are summarized in Table 3 ▸. The C—H hydrogen atoms were treated with calculated positions (methyl H atoms were allowed to rotate but not to tip) and were refined with *U*_iso_(H) = 1.2*U*_eq_(C) (1.5 for methyl H atoms) using a riding model with C—H = 0.95 Å for aromatic and 0.98 Å for methyl H atoms. The N—H and O—H hydrogen atoms were located in a difference map. Their bond lengths were set to ideal values, and finally they were refined with fixed bond lengths of N—H = 0.88 Å and O—H = 0.84 Å with *U*_iso_(H) = 1.5*U*_eq_(O,N) using a riding model. The methyl group of the methanol mol­ecule is disordered over two sets of sites and was refined using a split model with restraints for the bond lengths (SADI). After initial refinement of the s.o.f. it was fixed at 60:40 in the final refinement cycles. There were two weak residual electron density peaks that are located near centres of inversion, indicating for a disordered methanol solvent mol­ecule. However, a reasonable structural model could not be refined and therefore the contribution of this mol­ecule to the electronic density data was removed with the SQUEEZE proc­edure in *PLATON* (Spek, 2015 ▸). The volume of the solvent-accessible voids amounts to 68.7 Å^3^, and the number of electrons within the voids to 16.2, indicating that one methanol mol­ecule per formula unit is present. The given chemical formula and other crystal data take into account this methanol solvent mol­ecule.

## Supplementary Material

Crystal structure: contains datablock(s) I. DOI: 10.1107/S205698901801099X/wm5458sup1.cif

Structure factors: contains datablock(s) I. DOI: 10.1107/S205698901801099X/wm5458Isup2.hkl

CCDC reference: 1859612

Additional supporting information:  crystallographic information; 3D view; checkCIF report

## Figures and Tables

**Table 1:** Selected geometric parameters (Å, °)

Zn1—N4	2.050 (2)	Zn1—N2	2.0596 (19)
Zn1—N3	2.051 (2)	Zn1—O1	2.143 (2)
Zn1—N1	2.060 (2)		
			
N4—Zn1—N3	89.73 (8)	N1—Zn1—N2	88.96 (8)
N4—Zn1—N1	89.39 (8)	N4—Zn1—O1	92.93 (8)
N3—Zn1—N1	164.63 (8)	N3—Zn1—O1	98.66 (9)
N4—Zn1—N2	169.14 (8)	N1—Zn1—O1	96.70 (9)
N3—Zn1—N2	89.03 (8)	N2—Zn1—O1	97.93 (8)

**Table 2:** Hydrogen-bond geometry (Å, °)

*D*—H⋯*A*	*D*—H	H⋯*A*	*D*⋯*A*	*D*—H⋯*A*
C18—H18⋯Cl3^i^	0.95	2.97	3.858 (3)	157
N31—H31*A*⋯N41^ii^	0.88	2.63	3.318 (4)	136
N41—H41*B*⋯N21^ii^	0.88	2.61	3.437 (4)	156
O1—H1*O*1⋯N31^ii^	0.84	2.01	2.818 (3)	162
C61—H61*C*⋯N2	0.98	2.68	3.256 (8)	118
C61′—H61*F*⋯N1	0.98	2.59	3.292 (12)	129
C71—H71⋯N2	1.00	2.62	3.408 (4)	135

Symmetry codes: (i) 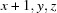; (ii) 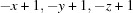.

**Table 3:** Experimental details

Crystal data	
Chemical formula	[Zn(C_44_H_32_N_8_)(CH_4_O)]·CHCl_3_·CH_4_O
*M*_r_	921.60
Crystal system, space group	Triclinic, *P* 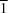
Temperature (K)	200
*a*, *b*, *c* (Å)	12.3880 (4), 13.2971 (4), 13.3656 (5)
α, β, γ (°)	90.159 (3), 110.550 (2), 90.800 (2)
*V* (Å^3^)	2061.27 (12)
*Z*	2
Radiation type	Mo *K*α
μ (mm^−1^)	0.84
Crystal size (mm)	0.20 × 0.10 × 0.08

Data collection	
Diffractometer	Stoe IPDS2
No. of measured, independent and observed [*I* > 2σ(*I*)] reflections	20461, 8071, 7001
*R*_int_	0.061
(sin θ/λ)_max_ (Å^−1^)	0.617

Refinement	
*R*[*F* ^2^ > 2σ(*F* ^2^)], *wR*(*F* ^2^), *S*	0.049, 0.138, 1.06
No. of reflections	8071
No. of parameters	543
No. of restraints	1
H-atom treatment	H-atom parameters constrained
Δρ_max_, Δρ_min_ (e Å^−3^)	0.65, −0.72

Computer programs: *X-AREA* (Stoe & Cie, 2008 ▸), *SHELXS97* (Sheldrick, 2008 ▸), *SHELXL2014* (Sheldrick, 2015 ▸), *XP* (Sheldrick, 2008 ▸), *DIAMOND* (Brandenburg, 2014 ▸) and *publCIF* (Westrip, 2010 ▸).

## supplementary crystallographic information

### Crystal data

**Table:**

[Zn(C_44_H_32_N_8_)(CH_4_O)]·CHCl_3_·CH_4_O	*Z* = 2
*M**_r_* = 921.60	*F*(000) = 916
Triclinic, *P*1	*D*_x_ = 1.485 Mg m^−^^3^
*a* = 12.3880 (4) Å	Mo *K*α radiation, λ = 0.71073 Å
*b* = 13.2971 (4) Å	Cell parameters from 20461 reflections
*c* = 13.3656 (5) Å	θ = 1.5–26.0°
α = 90.159 (3)°	µ = 0.84 mm^−^^1^
β = 110.550 (2)°	*T* = 200 K
γ = 90.800 (2)°	Block, red
*V* = 2061.27 (12) Å^3^	0.20 × 0.10 × 0.08 mm

### Data collection

**Table:**

Stoe IPDS-2 diffractometer	*R*_int_ = 0.061
ω scans	θ_max_ = 26.0°, θ_min_ = 1.5°
20461 measured reflections	*h* = −14→15
8071 independent reflections	*k* = −16→16
7001 reflections with *I* > 2σ(*I*)	*l* = −16→13

### Refinement

**Table:**

Refinement on *F*^2^	1 restraint
Least-squares matrix: full	Hydrogen site location: mixed
*R*[*F*^2^ > 2σ(*F*^2^)] = 0.049	H-atom parameters constrained
*wR*(*F*^2^) = 0.138	*w* = 1/[σ^2^(*F*_o_^2^) + (0.0778*P*)^2^ + 0.8654*P*] where *P* = (*F*_o_^2^ + 2*F*_c_^2^)/3
*S* = 1.06	(Δ/σ)_max_ = 0.018
8071 reflections	Δρ_max_ = 0.65 e Å^−^^3^
543 parameters	Δρ_min_ = −0.71 e Å^−^^3^

### Special details

**Table:**

Geometry. All esds (except the esd in the dihedral angle between two l.s. planes) are estimated using the full covariance matrix. The cell esds are taken into account individually in the estimation of esds in distances, angles and torsion angles; correlations between esds in cell parameters are only used when they are defined by crystal symmetry. An approximate (isotropic) treatment of cell esds is used for estimating esds involving l.s. planes.

### Fractional atomic coordinates and isotropic or equivalent isotropic displacement parameters (Å^2^)

**Table:**

	*x*	*y*	*z*	*U*_iso_*/*U*_eq_	Occ. (<1)
Zn1	0.43236 (2)	0.35668 (2)	0.26068 (2)	0.03716 (11)	
N1	0.45369 (17)	0.20660 (15)	0.23529 (17)	0.0392 (4)	
N2	0.29092 (16)	0.31816 (15)	0.30033 (16)	0.0374 (4)	
N3	0.37962 (17)	0.50321 (15)	0.24521 (17)	0.0385 (4)	
N4	0.55193 (17)	0.39265 (16)	0.19196 (17)	0.0401 (4)	
C1	0.5373 (2)	0.16696 (18)	0.2019 (2)	0.0398 (5)	
C2	0.5314 (2)	0.05862 (19)	0.2046 (2)	0.0439 (6)	
H2	0.5786	0.0131	0.1843	0.053*	
C3	0.4461 (2)	0.03407 (19)	0.2416 (2)	0.0430 (5)	
H3	0.4225	−0.0320	0.2526	0.052*	
C4	0.3976 (2)	0.12672 (18)	0.26130 (19)	0.0375 (5)	
C5	0.3088 (2)	0.13389 (18)	0.3032 (2)	0.0387 (5)	
C6	0.2598 (2)	0.22319 (18)	0.32034 (19)	0.0383 (5)	
C7	0.1638 (2)	0.2292 (2)	0.3573 (2)	0.0441 (6)	
H7	0.1263	0.1743	0.3776	0.053*	
C8	0.1370 (2)	0.3273 (2)	0.3576 (2)	0.0436 (6)	
H8	0.0764	0.3541	0.3772	0.052*	
C9	0.2173 (2)	0.38384 (19)	0.3227 (2)	0.0385 (5)	
C10	0.2165 (2)	0.48791 (19)	0.3095 (2)	0.0391 (5)	
C11	0.2886 (2)	0.54284 (18)	0.2684 (2)	0.0385 (5)	
C12	0.2830 (2)	0.64903 (19)	0.2469 (2)	0.0438 (6)	
H12	0.2270	0.6938	0.2541	0.053*	
C13	0.3717 (2)	0.6735 (2)	0.2145 (2)	0.0446 (6)	
H13	0.3901	0.7385	0.1953	0.053*	
C14	0.4331 (2)	0.58211 (18)	0.2149 (2)	0.0389 (5)	
C15	0.5354 (2)	0.57684 (19)	0.1915 (2)	0.0398 (5)	
C16	0.5888 (2)	0.48839 (19)	0.1807 (2)	0.0409 (5)	
C17	0.6925 (2)	0.4819 (2)	0.1543 (3)	0.0505 (6)	
H17	0.7369	0.5369	0.1435	0.061*	
C18	0.7142 (2)	0.3837 (2)	0.1477 (3)	0.0535 (7)	
H18	0.7762	0.3566	0.1306	0.064*	
C19	0.6263 (2)	0.32731 (19)	0.1714 (2)	0.0423 (5)	
C20	0.6187 (2)	0.22237 (19)	0.1735 (2)	0.0417 (5)	
C21	0.2652 (2)	0.03777 (19)	0.3352 (2)	0.0433 (6)	
C22	0.3084 (3)	0.0086 (2)	0.4418 (3)	0.0590 (7)	
N21	0.3864 (3)	0.0707 (2)	0.5193 (2)	0.0769 (9)	
H21A	0.4285	0.1145	0.4991	0.115*	
H21B	0.4184	0.0328	0.5755	0.115*	
C23	0.2686 (5)	−0.0817 (3)	0.4704 (3)	0.0911 (14)	
H23	0.2979	−0.1031	0.5426	0.109*	
C24	0.1869 (4)	−0.1403 (3)	0.3946 (4)	0.0908 (14)	
H24	0.1611	−0.2018	0.4153	0.109*	
C25	0.1428 (3)	−0.1111 (2)	0.2904 (3)	0.0672 (9)	
H25	0.0855	−0.1511	0.2391	0.081*	
C26	0.1828 (2)	−0.0221 (2)	0.2602 (3)	0.0515 (6)	
H26	0.1536	−0.0020	0.1875	0.062*	
C31	0.1304 (2)	0.54603 (18)	0.3423 (2)	0.0402 (5)	
C32	0.1437 (2)	0.55734 (18)	0.4504 (2)	0.0410 (5)	
N31	0.2392 (2)	0.51753 (18)	0.53272 (18)	0.0490 (5)	
H31A	0.2580	0.4572	0.5179	0.074*	
H31B	0.2298	0.5134	0.5949	0.074*	
C33	0.0628 (2)	0.6129 (2)	0.4776 (2)	0.0478 (6)	
H33	0.0716	0.6213	0.5507	0.057*	
C34	−0.0293 (2)	0.6555 (2)	0.4001 (3)	0.0533 (7)	
H34	−0.0836	0.6927	0.4200	0.064*	
C35	−0.0434 (2)	0.6447 (2)	0.2934 (3)	0.0536 (7)	
H35	−0.1067	0.6747	0.2398	0.064*	
C36	0.0363 (2)	0.5893 (2)	0.2655 (2)	0.0478 (6)	
H36	0.0262	0.5810	0.1922	0.057*	
C41	0.5929 (2)	0.67416 (19)	0.1812 (2)	0.0443 (6)	
C42	0.6476 (2)	0.7341 (2)	0.2715 (2)	0.0486 (6)	
N41	0.6512 (2)	0.7034 (2)	0.3725 (2)	0.0605 (6)	
H41A	0.5894	0.6694	0.3714	0.091*	
H41B	0.6646	0.7584	0.4120	0.091*	
C43	0.7059 (3)	0.8215 (2)	0.2602 (3)	0.0645 (9)	
H43	0.7443	0.8622	0.3212	0.077*	
C44	0.7086 (3)	0.8498 (2)	0.1621 (4)	0.0687 (10)	
H44	0.7487	0.9096	0.1562	0.082*	
C45	0.6540 (3)	0.7924 (3)	0.0732 (3)	0.0661 (9)	
H45	0.6552	0.8126	0.0054	0.079*	
C46	0.5969 (2)	0.7043 (2)	0.0828 (3)	0.0539 (7)	
H46	0.5598	0.6639	0.0211	0.065*	
C51	0.7054 (2)	0.16490 (19)	0.1419 (2)	0.0426 (5)	
C52	0.8152 (2)	0.1493 (2)	0.2161 (2)	0.0533 (7)	
N51	0.8467 (3)	0.1884 (3)	0.3194 (3)	0.0885 (11)	
H51A	0.7907	0.2197	0.3322	0.133*	
H51B	0.9128	0.1683	0.3654	0.133*	
C53	0.8949 (2)	0.0959 (2)	0.1844 (3)	0.0591 (8)	
H53	0.9696	0.0841	0.2348	0.071*	
C54	0.8665 (3)	0.0608 (2)	0.0824 (3)	0.0559 (7)	
H54	0.9222	0.0266	0.0617	0.067*	
C55	0.7573 (3)	0.0747 (3)	0.0091 (3)	0.0645 (8)	
H55	0.7368	0.0487	−0.0615	0.077*	
C56	0.6776 (3)	0.1271 (3)	0.0397 (2)	0.0569 (7)	
H56	0.6024	0.1370	−0.0108	0.068*	
O1	0.55695 (18)	0.36589 (17)	0.41938 (17)	0.0589 (5)	
H1O1	0.6091	0.4078	0.4214	0.088*	
C61	0.5284 (8)	0.3528 (12)	0.5039 (5)	0.148 (6)	0.6
H61A	0.5033	0.4168	0.5242	0.222*	0.6
H61B	0.5952	0.3288	0.5629	0.222*	0.6
H61C	0.4654	0.3030	0.4877	0.222*	0.6
C61'	0.5955 (13)	0.2855 (9)	0.4808 (9)	0.113 (5)	0.4
H61D	0.5594	0.2819	0.5352	0.169*	0.4
H61E	0.6794	0.2912	0.5157	0.169*	0.4
H61F	0.5761	0.2245	0.4364	0.169*	0.4
C71	0.0752 (3)	0.2987 (3)	0.0578 (3)	0.0622 (8)	
H71	0.0983	0.2929	0.1371	0.075*	
Cl1	0.16172 (10)	0.39208 (8)	0.02947 (8)	0.0849 (3)	
Cl2	0.09745 (11)	0.18359 (8)	0.00662 (12)	0.0989 (4)	
Cl3	−0.07064 (10)	0.32776 (12)	0.00537 (12)	0.1104 (5)	

### Atomic displacement parameters (Å^2^)

**Table:**

	*U*^11^	*U*^22^	*U*^33^	*U*^12^	*U*^13^	*U*^23^
Zn1	0.03244 (16)	0.03754 (17)	0.04684 (18)	−0.00091 (11)	0.02067 (12)	−0.00504 (11)
N1	0.0354 (10)	0.0391 (10)	0.0492 (11)	−0.0002 (8)	0.0225 (9)	−0.0044 (8)
N2	0.0314 (9)	0.0389 (10)	0.0462 (11)	0.0003 (8)	0.0191 (8)	−0.0057 (8)
N3	0.0334 (10)	0.0386 (10)	0.0485 (11)	−0.0012 (8)	0.0206 (9)	−0.0061 (8)
N4	0.0362 (10)	0.0392 (10)	0.0516 (12)	−0.0008 (8)	0.0239 (9)	−0.0050 (9)
C1	0.0373 (12)	0.0396 (12)	0.0479 (13)	0.0041 (9)	0.0216 (10)	−0.0033 (10)
C2	0.0410 (13)	0.0415 (13)	0.0552 (15)	0.0041 (10)	0.0242 (11)	−0.0044 (11)
C3	0.0420 (13)	0.0378 (12)	0.0529 (14)	−0.0003 (10)	0.0212 (11)	−0.0046 (10)
C4	0.0347 (11)	0.0371 (12)	0.0431 (12)	−0.0009 (9)	0.0166 (10)	−0.0033 (9)
C5	0.0363 (12)	0.0402 (12)	0.0429 (12)	−0.0033 (9)	0.0181 (10)	−0.0046 (10)
C6	0.0331 (11)	0.0413 (12)	0.0433 (12)	−0.0048 (9)	0.0174 (10)	−0.0067 (10)
C7	0.0397 (13)	0.0463 (13)	0.0540 (14)	−0.0060 (10)	0.0264 (11)	−0.0067 (11)
C8	0.0383 (13)	0.0455 (13)	0.0549 (15)	−0.0025 (10)	0.0262 (11)	−0.0087 (11)
C9	0.0320 (11)	0.0447 (13)	0.0439 (12)	−0.0009 (9)	0.0198 (10)	−0.0071 (10)
C10	0.0328 (11)	0.0430 (13)	0.0437 (13)	−0.0001 (9)	0.0163 (10)	−0.0082 (10)
C11	0.0318 (11)	0.0405 (12)	0.0456 (13)	0.0015 (9)	0.0167 (10)	−0.0079 (10)
C12	0.0379 (13)	0.0410 (13)	0.0556 (15)	0.0036 (10)	0.0202 (11)	−0.0022 (11)
C13	0.0384 (13)	0.0400 (13)	0.0576 (15)	0.0016 (10)	0.0196 (11)	−0.0013 (11)
C14	0.0358 (12)	0.0372 (12)	0.0463 (13)	−0.0015 (9)	0.0176 (10)	−0.0042 (10)
C15	0.0367 (12)	0.0410 (12)	0.0453 (13)	−0.0019 (10)	0.0188 (10)	−0.0021 (10)
C16	0.0362 (12)	0.0416 (12)	0.0506 (14)	−0.0028 (10)	0.0223 (11)	−0.0023 (10)
C17	0.0440 (14)	0.0462 (14)	0.0739 (18)	−0.0022 (11)	0.0365 (14)	−0.0016 (13)
C18	0.0468 (15)	0.0487 (15)	0.081 (2)	0.0004 (12)	0.0420 (15)	−0.0026 (13)
C19	0.0371 (12)	0.0433 (13)	0.0563 (14)	0.0015 (10)	0.0286 (11)	−0.0045 (11)
C20	0.0365 (12)	0.0450 (13)	0.0504 (14)	0.0031 (10)	0.0239 (11)	−0.0059 (10)
C21	0.0423 (13)	0.0392 (12)	0.0569 (15)	−0.0039 (10)	0.0281 (12)	−0.0062 (11)
C22	0.076 (2)	0.0516 (16)	0.0580 (17)	−0.0103 (14)	0.0345 (16)	−0.0018 (13)
N21	0.104 (2)	0.0721 (19)	0.0483 (14)	−0.0158 (17)	0.0192 (15)	−0.0013 (13)
C23	0.141 (4)	0.071 (2)	0.075 (2)	−0.028 (3)	0.056 (3)	0.0063 (19)
C24	0.128 (4)	0.058 (2)	0.112 (3)	−0.031 (2)	0.075 (3)	−0.005 (2)
C25	0.0640 (19)	0.0487 (16)	0.099 (3)	−0.0172 (14)	0.0425 (19)	−0.0192 (17)
C26	0.0451 (14)	0.0445 (14)	0.0709 (18)	−0.0032 (11)	0.0281 (13)	−0.0087 (13)
C31	0.0334 (12)	0.0415 (12)	0.0510 (14)	−0.0007 (9)	0.0218 (10)	−0.0084 (10)
C32	0.0386 (12)	0.0382 (12)	0.0527 (14)	−0.0041 (10)	0.0242 (11)	−0.0068 (10)
N31	0.0502 (13)	0.0534 (13)	0.0479 (12)	−0.0004 (10)	0.0228 (10)	−0.0025 (10)
C33	0.0481 (15)	0.0474 (14)	0.0598 (16)	−0.0040 (11)	0.0341 (13)	−0.0086 (12)
C34	0.0466 (15)	0.0466 (14)	0.080 (2)	0.0024 (11)	0.0388 (15)	−0.0082 (13)
C35	0.0370 (13)	0.0549 (16)	0.0708 (19)	0.0050 (11)	0.0213 (13)	−0.0020 (14)
C36	0.0387 (13)	0.0532 (15)	0.0533 (15)	0.0029 (11)	0.0183 (11)	−0.0050 (12)
C41	0.0344 (12)	0.0395 (13)	0.0637 (16)	0.0017 (10)	0.0232 (11)	0.0013 (11)
C42	0.0410 (13)	0.0389 (13)	0.0684 (17)	0.0023 (10)	0.0224 (12)	−0.0038 (12)
N41	0.0587 (15)	0.0572 (15)	0.0633 (16)	−0.0062 (12)	0.0191 (12)	−0.0145 (12)
C43	0.0491 (16)	0.0405 (14)	0.107 (3)	−0.0032 (12)	0.0312 (17)	−0.0127 (16)
C44	0.0558 (18)	0.0471 (16)	0.118 (3)	0.0042 (13)	0.048 (2)	0.0154 (18)
C45	0.0544 (17)	0.0641 (19)	0.093 (2)	0.0103 (15)	0.0413 (18)	0.0259 (18)
C46	0.0452 (15)	0.0586 (16)	0.0649 (17)	0.0034 (12)	0.0279 (13)	0.0112 (14)
C51	0.0384 (12)	0.0404 (12)	0.0587 (15)	0.0009 (10)	0.0293 (12)	−0.0051 (11)
C52	0.0422 (14)	0.0596 (17)	0.0639 (17)	0.0020 (12)	0.0259 (13)	−0.0129 (13)
N51	0.0580 (17)	0.133 (3)	0.0669 (18)	0.0229 (18)	0.0125 (14)	−0.0369 (19)
C53	0.0357 (14)	0.0639 (18)	0.079 (2)	0.0021 (12)	0.0223 (14)	−0.0158 (15)
C54	0.0490 (15)	0.0497 (15)	0.084 (2)	−0.0022 (12)	0.0419 (15)	−0.0143 (14)
C55	0.0613 (19)	0.079 (2)	0.0621 (18)	0.0080 (16)	0.0331 (16)	−0.0169 (16)
C56	0.0487 (15)	0.0716 (19)	0.0554 (16)	0.0109 (14)	0.0245 (13)	−0.0073 (14)
O1	0.0474 (11)	0.0686 (13)	0.0561 (12)	−0.0119 (10)	0.0130 (9)	−0.0004 (10)
C61	0.082 (6)	0.316 (17)	0.046 (4)	−0.090 (8)	0.027 (4)	−0.023 (6)
C61'	0.133 (12)	0.112 (10)	0.057 (6)	−0.060 (9)	−0.011 (7)	0.025 (6)
C71	0.069 (2)	0.0665 (19)	0.0525 (17)	0.0045 (15)	0.0230 (15)	0.0008 (14)
Cl1	0.0959 (7)	0.0763 (6)	0.0706 (5)	−0.0257 (5)	0.0152 (5)	−0.0023 (4)
Cl2	0.1039 (8)	0.0724 (6)	0.1507 (11)	−0.0125 (5)	0.0832 (8)	−0.0244 (6)
Cl3	0.0809 (7)	0.1427 (11)	0.1283 (10)	0.0452 (7)	0.0606 (7)	0.0528 (9)

### Geometric parameters (Å, º)

**Table:**

Zn1—N4	2.050 (2)	C25—C26	1.391 (4)
Zn1—N3	2.051 (2)	C25—H25	0.9500
Zn1—N1	2.060 (2)	C26—H26	0.9500
Zn1—N2	2.0596 (19)	C31—C36	1.387 (4)
Zn1—O1	2.143 (2)	C31—C32	1.403 (4)
N1—C4	1.372 (3)	C32—C33	1.399 (3)
N1—C1	1.374 (3)	C32—N31	1.413 (4)
N2—C6	1.370 (3)	N31—H31A	0.8801
N2—C9	1.378 (3)	N31—H31B	0.8799
N3—C14	1.370 (3)	C33—C34	1.374 (4)
N3—C11	1.381 (3)	C33—H33	0.9500
N4—C19	1.371 (3)	C34—C35	1.381 (4)
N4—C16	1.373 (3)	C34—H34	0.9500
C1—C20	1.397 (3)	C35—C36	1.391 (4)
C1—C2	1.443 (4)	C35—H35	0.9500
C2—C3	1.351 (4)	C36—H36	0.9500
C2—H2	0.9500	C41—C46	1.394 (4)
C3—C4	1.441 (3)	C41—C42	1.399 (4)
C3—H3	0.9500	C42—C43	1.396 (4)
C4—C5	1.403 (3)	C42—N41	1.397 (4)
C5—C6	1.396 (3)	N41—H41A	0.8800
C5—C21	1.501 (3)	N41—H41B	0.8800
C6—C7	1.442 (3)	C43—C44	1.376 (5)
C7—C8	1.351 (4)	C43—H43	0.9500
C7—H7	0.9500	C44—C45	1.367 (6)
C8—C9	1.442 (3)	C44—H44	0.9500
C8—H8	0.9500	C45—C46	1.390 (4)
C9—C10	1.395 (4)	C45—H45	0.9500
C10—C11	1.401 (3)	C46—H46	0.9500
C10—C31	1.509 (3)	C51—C56	1.377 (4)
C11—C12	1.439 (4)	C51—C52	1.393 (4)
C12—C13	1.351 (4)	C52—N51	1.393 (4)
C12—H12	0.9500	C52—C53	1.403 (4)
C13—C14	1.442 (3)	N51—H51A	0.8801
C13—H13	0.9500	N51—H51B	0.8800
C14—C15	1.410 (3)	C53—C54	1.362 (5)
C15—C16	1.389 (4)	C53—H53	0.9500
C15—C41	1.498 (3)	C54—C55	1.379 (5)
C16—C17	1.451 (3)	C54—H54	0.9500
C17—C18	1.346 (4)	C55—C56	1.389 (4)
C17—H17	0.9500	C55—H55	0.9500
C18—C19	1.438 (3)	C56—H56	0.9500
C18—H18	0.9500	O1—C61	1.307 (6)
C19—C20	1.398 (4)	O1—C61'	1.337 (10)
C20—C51	1.502 (3)	O1—H1O1	0.8400
C21—C26	1.391 (4)	C61—H61A	0.9800
C21—C22	1.393 (4)	C61—H61B	0.9800
C22—C23	1.396 (5)	C61—H61C	0.9800
C22—N21	1.399 (4)	C61'—H61D	0.9800
N21—H21A	0.8800	C61'—H61E	0.9800
N21—H21B	0.8800	C61'—H61F	0.9800
C23—C24	1.382 (6)	C71—Cl2	1.739 (4)
C23—H23	0.9500	C71—Cl3	1.742 (4)
C24—C25	1.365 (6)	C71—Cl1	1.757 (4)
C24—H24	0.9500	C71—H71	1.0000
			
N4—Zn1—N3	89.73 (8)	C25—C24—H24	119.5
N4—Zn1—N1	89.39 (8)	C23—C24—H24	119.5
N3—Zn1—N1	164.63 (8)	C24—C25—C26	119.2 (3)
N4—Zn1—N2	169.14 (8)	C24—C25—H25	120.4
N3—Zn1—N2	89.03 (8)	C26—C25—H25	120.4
N1—Zn1—N2	88.96 (8)	C25—C26—C21	120.7 (3)
N4—Zn1—O1	92.93 (8)	C25—C26—H26	119.6
N3—Zn1—O1	98.66 (9)	C21—C26—H26	119.6
N1—Zn1—O1	96.70 (9)	C36—C31—C32	118.8 (2)
N2—Zn1—O1	97.93 (8)	C36—C31—C10	120.3 (2)
C4—N1—C1	106.7 (2)	C32—C31—C10	120.9 (2)
C4—N1—Zn1	126.70 (16)	C33—C32—C31	119.1 (2)
C1—N1—Zn1	126.15 (16)	C33—C32—N31	119.1 (2)
C6—N2—C9	107.12 (19)	C31—C32—N31	121.7 (2)
C6—N2—Zn1	126.29 (16)	C32—N31—H31A	113.3
C9—N2—Zn1	126.26 (16)	C32—N31—H31B	114.1
C14—N3—C11	106.6 (2)	H31A—N31—H31B	106.7
C14—N3—Zn1	125.82 (16)	C34—C33—C32	120.9 (3)
C11—N3—Zn1	127.46 (16)	C34—C33—H33	119.5
C19—N4—C16	107.4 (2)	C32—C33—H33	119.5
C19—N4—Zn1	126.01 (17)	C33—C34—C35	120.5 (2)
C16—N4—Zn1	125.19 (16)	C33—C34—H34	119.8
N1—C1—C20	125.6 (2)	C35—C34—H34	119.8
N1—C1—C2	109.5 (2)	C34—C35—C36	119.0 (3)
C20—C1—C2	124.9 (2)	C34—C35—H35	120.5
C3—C2—C1	107.1 (2)	C36—C35—H35	120.5
C3—C2—H2	126.5	C31—C36—C35	121.6 (3)
C1—C2—H2	126.5	C31—C36—H36	119.2
C2—C3—C4	107.3 (2)	C35—C36—H36	119.2
C2—C3—H3	126.4	C46—C41—C42	119.0 (3)
C4—C3—H3	126.4	C46—C41—C15	120.7 (3)
N1—C4—C5	125.4 (2)	C42—C41—C15	120.2 (2)
N1—C4—C3	109.5 (2)	C43—C42—N41	120.4 (3)
C5—C4—C3	125.1 (2)	C43—C42—C41	118.8 (3)
C6—C5—C4	125.4 (2)	N41—C42—C41	120.6 (2)
C6—C5—C21	117.3 (2)	C42—N41—H41A	113.6
C4—C5—C21	117.3 (2)	C42—N41—H41B	105.8
N2—C6—C5	125.9 (2)	H41A—N41—H41B	113.8
N2—C6—C7	109.2 (2)	C44—C43—C42	121.2 (3)
C5—C6—C7	124.8 (2)	C44—C43—H43	119.4
C8—C7—C6	107.4 (2)	C42—C43—H43	119.4
C8—C7—H7	126.3	C45—C44—C43	120.5 (3)
C6—C7—H7	126.3	C45—C44—H44	119.8
C7—C8—C9	107.3 (2)	C43—C44—H44	119.8
C7—C8—H8	126.3	C44—C45—C46	119.4 (3)
C9—C8—H8	126.3	C44—C45—H45	120.3
N2—C9—C10	125.9 (2)	C46—C45—H45	120.3
N2—C9—C8	109.0 (2)	C45—C46—C41	121.1 (3)
C10—C9—C8	125.1 (2)	C45—C46—H46	119.4
C9—C10—C11	125.6 (2)	C41—C46—H46	119.4
C9—C10—C31	117.0 (2)	C56—C51—C52	119.3 (2)
C11—C10—C31	117.3 (2)	C56—C51—C20	120.7 (2)
N3—C11—C10	124.7 (2)	C52—C51—C20	120.0 (2)
N3—C11—C12	109.1 (2)	C51—C52—N51	120.6 (3)
C10—C11—C12	126.2 (2)	C51—C52—C53	118.9 (3)
C13—C12—C11	107.6 (2)	N51—C52—C53	120.4 (3)
C13—C12—H12	126.2	C52—N51—H51A	113.9
C11—C12—H12	126.2	C52—N51—H51B	116.3
C12—C13—C14	106.9 (2)	H51A—N51—H51B	128.5
C12—C13—H13	126.5	C54—C53—C52	120.9 (3)
C14—C13—H13	126.5	C54—C53—H53	119.5
N3—C14—C15	125.8 (2)	C52—C53—H53	119.5
N3—C14—C13	109.7 (2)	C53—C54—C55	120.3 (3)
C15—C14—C13	124.5 (2)	C53—C54—H54	119.8
C16—C15—C14	125.0 (2)	C55—C54—H54	119.8
C16—C15—C41	117.6 (2)	C54—C55—C56	119.2 (3)
C14—C15—C41	117.4 (2)	C54—C55—H55	120.4
N4—C16—C15	126.0 (2)	C56—C55—H55	120.4
N4—C16—C17	108.6 (2)	C51—C56—C55	121.3 (3)
C15—C16—C17	125.5 (2)	C51—C56—H56	119.4
C18—C17—C16	107.4 (2)	C55—C56—H56	119.4
C18—C17—H17	126.3	C61—O1—Zn1	122.0 (4)
C16—C17—H17	126.3	C61'—O1—Zn1	123.3 (5)
C17—C18—C19	107.4 (2)	C61—O1—H1O1	119.7
C17—C18—H18	126.3	C61'—O1—H1O1	113.6
C19—C18—H18	126.3	Zn1—O1—H1O1	109.9
N4—C19—C20	125.6 (2)	O1—C61—H61A	109.5
N4—C19—C18	109.2 (2)	O1—C61—H61B	109.5
C20—C19—C18	125.2 (2)	H61A—C61—H61B	109.5
C1—C20—C19	125.6 (2)	O1—C61—H61C	109.5
C1—C20—C51	117.6 (2)	H61A—C61—H61C	109.5
C19—C20—C51	116.8 (2)	H61B—C61—H61C	109.5
C26—C21—C22	119.9 (3)	O1—C61'—H61D	109.5
C26—C21—C5	121.0 (3)	O1—C61'—H61E	109.5
C22—C21—C5	119.1 (2)	H61D—C61'—H61E	109.5
C21—C22—C23	118.6 (3)	O1—C61'—H61F	109.5
C21—C22—N21	120.6 (3)	H61D—C61'—H61F	109.5
C23—C22—N21	120.8 (3)	H61E—C61'—H61F	109.5
C22—N21—H21A	118.7	Cl2—C71—Cl3	109.7 (2)
C22—N21—H21B	106.3	Cl2—C71—Cl1	109.77 (19)
H21A—N21—H21B	120.3	Cl3—C71—Cl1	111.85 (19)
C24—C23—C22	120.6 (4)	Cl2—C71—H71	108.5
C24—C23—H23	119.7	Cl3—C71—H71	108.5
C22—C23—H23	119.7	Cl1—C71—H71	108.5
C25—C24—C23	120.9 (3)		

### Hydrogen-bond geometry (Å, º)

**Table:**

*D*—H···*A*	*D*—H	H···*A*	*D*···*A*	*D*—H···*A*
C18—H18···Cl3^i^	0.95	2.97	3.858 (3)	157
N31—H31*A*···N41^ii^	0.88	2.63	3.318 (4)	136
N41—H41*B*···N21^ii^	0.88	2.61	3.437 (4)	156
O1—H1*O*1···N31^ii^	0.84	2.01	2.818 (3)	162
C61—H61*C*···N2	0.98	2.68	3.256 (8)	118
C61′—H61*F*···N1	0.98	2.59	3.292 (12)	129
C71—H71···N2	1.00	2.62	3.408 (4)	135

Symmetry codes: (i) *x*+1, *y*, *z*; (ii) −*x*+1, −*y*+1, −*z*+1.
